# Predictive role of MGMT and IDH status in the efficacy of bevacizumab for high-grade glioma: a retrospective study

**DOI:** 10.3389/fonc.2025.1634892

**Published:** 2025-08-25

**Authors:** Bai Xuexue, Yan Chengrui, Wenbin Ma, Feng Ming, Wu Chao

**Affiliations:** ^1^ Department of Neurosurgery, Peking Union Medical College Hospital, Chinese Academy of Medical Science and Peking Union Medical College, Beijing, China; ^2^ Department of Neurosurgery, Tengzhou Central People’s Hospital, Tengzhou, Shandong, China

**Keywords:** bevacizumab, high-grade glioma, MGMT, IDH, predictor

## Abstract

**Background:**

The objective of this study is to investigate the predictive role of O6-methylguanine-DNA methyltransferase (MGMT) and isocitrate dehydrogenase (IDH) status on the efficacy of bevacizumab (BEV) in high-grade glioma (HGG), while excluding the interference of chemotherapy agents.

**Methods:**

A retrospective, single-center analysis was conducted on 103 patients with HGG who received BEV treatment. The enrolled patients were grouped based on their different biomarker statuses. Depending on whether the numerical variables of the patients satisfied the normal distribution, t-test or rank-sum test was employed. Chi-square test was used for the comparison of categorical variables. Univariate and multivariate Cox regression analyses were performed to identify prognostic factors affecting progression-free survival (PFS) and overall survival (OS).

**Results:**

Multivariate COX regression analysis revealed that pathological grade, extent of resection, MGMT status, and IDH status were independent factors influencing PFS and OS in patients with HGG. The PFS, OS, and therapeutic response were superior in the MGMT methylated group compared to the unmethylated group. Similarly, patients with IDH mutations exhibited better PFS, OS, and therapeutic response than those with IDH wild-type.

**Conclusions:**

After controlling for potential confounding effects of chemotherapeutic agents, HGG patients with concurrent MGMT methylation and IDH mutations are likely to derive greater benefit from BEV treatment.

## 1 Introduction

High-grade gliomas (HGG) represent the most common and fatal type of primary intracranial tumor (1). Despite ongoing efforts to improve outcomes related to HGG, their prognosis remains dismal (2). Prior to the release of the World Health Organization’s (WHO) fifth edition of the Classification of Tumors of the Central Nervous System (CNS) in June 2021 (“WHO CNS5 Classification”), the classification of HGG primarily relied on histological features (3). However, the WHO CNS5 Classification scheme incorporated molecular information along with histological characteristics into a comprehensive diagnostic approach, significantly altering the overall categorization of gliomas (4). Notably, one of the most significant changes in adult diffuse gliomas was the reclassification of “GBM, isocitrate dehydrogenase (IDH) mutant, WHO grade 4” to “Astrocytoma, IDH-mutant, WHO grade 4” (5). IDH-mutant gliomas fundamentally differ from IDH-wildtype gliomas in terms of metabolism, epigenetics, biological behavior, invasive infiltration, susceptible populations, and therapeutic response (6).

The standard treatment for HGG typically involves surgical resection followed by concurrent chemoradiotherapy and subsequent adjuvant chemotherapy (7). Temozolomide (TMZ) is the most commonly used chemotherapeutic agent for HGG (8). In real-world settings, the use of TMZ is often limited by various reasons leading to discontinuation of therapy (9). Bevacizumab (BEV), a monoclonal antibody targeting vascular endothelial growth factor (VEGF), has emerged as a promising agent in the treatment paradigm for HGG, demonstrating the potential to prolong progression-free survival (PFS), although its impact on overall survival (OS) remains unclear. It is widely known that the therapeutic effects of chemotherapy drugs vary among HGG patients with different genotypes (10). Previous studies exploring the relationship between O6-methylguanine-DNA methyltransferase (MGMT) status and the efficacy of BEV have often been confounded by the interference of chemotherapy drugs, resulting in uncertainty regarding the association between BEV effectiveness and MGMT status (11). Furthermore, there is a lack of research exploring the relationship between BEV efficacy and IDH mutation status. In this retrospective study, we aimed to analyze the impact of MGMT and IDH status on the effectiveness of BEV, while excluding the confounding effects of chemotherapy drugs.

## 2 Material and methods

### 2.1 Enrolled patients

The inclusion criteria for this study were as follows: (1) patients with histologically confirmed grade 3 or 4 gliomas; (2) age greater than 18 years; (3) patients had not undergone BEV therapy prior to this study; and (4) patients with comprehensive clinical data available. The exclusion criteria included: (1) patients with a history of recent abnormal bleeding; (2) patients with other concurrent malignancies in addition to the primary glioma; and (3) patients with severe systemic diseases. All treatments were administered by the same neuro-oncology team following institutional protocol to ensure consistency in drug administration and monitoring. A total of 103 patients meeting these criteria were included in this study. All these patients had undergone surgery and radiotherapy, but had not received or had discontinued short-term TMZ treatment before starting BEV therapy. **Table 1** summarizes the detailed demographic characteristics of all patients.

**Table 1:** Baseline demographic characteristics of all patients.

Parameter	MGMT		P-value
	Methylation (n=59)	Unmethylation (n=44)	
Age (mean, years)	50.53 ± 10.94	51.32 ± 9.45	0.280
Sex (N)			0.978
Male	28	21	
Female	31	23	
Pathological grade (N)			0.412
WHO III	22	13	
WHO IV	37	31	
Extent of resection (N)			0.738
Gross resection	37	29	
Partial resection	22	15	
IDH (N)			0.404
Mutation	37	24	
Wild type	22	20	
Radiation therapy (N)			0.795
EBRT	39	28	
IMRT	20	16	
Tumor volume (ccm)	29.73 ± 10.90	27.70 ± 10.55	0.291
KPS	65.08 ± 12.78	63.86 ± 11.25	0.652
TMZ cycle	0.84 ± 0.58	0.81 ± 0.55	0.858
TMZ interval	2.08 ± 1.03	2.16 ± 1.13	0.840
Steroids (mg)	28.98 ± 11.70	25.23 ± 11.31	0.113

MGMT, O6-methylguanine-DNA methyltransferase; WHO, world health organization; IDH, isocitrate dehydrogenase; KPS, karnofsky performance score; TMZ, temozolomide; EBRT, Conventional External Beam Radiation Therapy; IMRT, Intensity-Modulated Radiation Therapy.

### 2.2 Treatment situation

All patients received radiotherapy at our institution, with 67 patients undergoing conventional external beam radiation therapy (EBRT) and the remaining 36 receiving intensity-modulated radiation therapy (IMRT). The EBRT regimen consisted of 30 fractions of 2 Gy each (total dose: 60 Gy). For IMRT, the prescribed dose was isotoxic to the EBRT protocol, with no significant deviations in total dose or fractionation among the 36 patients. Eleven patients did not receive TMZ treatment, while the remaining patients were administered the standard 5-day TMZ protocol with a cycle duration of 28 days (12). The maximum number of cycles of TMZ treatment in this study was two. Due to intolerable adverse reactions such as hematological toxicity and vomiting, these patients discontinued TMZ treatment after a short period and commenced BEV therapy. While previous studies have recommended a BEV dose range of 5 to 15 mg/kg, some reports have indicated that a dose of 5 mg/kg is both more effective and safer than 10 mg/kg (13). Therefore, all enrolled patients in this study received a BEV dose of 5 mg/kg, administered every two weeks.

### 2.3 Post-treatment evaluation

Imaging studies were completed within two days post-surgery, utilizing t1-weighted enhancement sequences to assess tumor status (14). Gross resection was defined as residual enhancement signals not exceeding 5% of the preoperative volume, while partial resection was defined as residual enhancement signals greater than 5% of the preoperative volume. Imaging studies were conducted every 1-2 months during the follow-up period (15). Therapeutic response was evaluated according to the Response Assessment in Neuro-Oncology (RANO) criteria (16). Complete response (CR) was defined as the disappearance of tumor signals, partial response (PR) as a reduction in tumor area by at least 50%, stable disease (SD) as a reduction in tumor size by less than 50% or an increase by less than 25%, and disease progression (PD) as an increase in tumor size by at least 25% (17). The overall response rate (OR) encompassed both CR and PR. PFS was defined as the time from the start of BEV therapy to the occurrence of tumor progression, whereas OS was defined as the time from the start of BEV therapy to death or the end of follow-up.

### 2.4 Statistical analysis

Continuous variables were expressed as mean ± standard deviation (mean ± SD), while categorical variables were represented by patient counts or percentages (%). If the continuous variables followed a normal distribution, the t-test was used for comparison; otherwise, the Mann-Whitney U test was employed. The chi-square test was applied for comparing categorical variables. Univariate and multivariate Cox regression models were utilized to investigate the independent risk factors for PFS and OS in HGG patients. Kaplan-Meier method was adopted to generate survival curves for PFS and OS. All data in this study were analyzed using SPSS (version 27.0, IBM). A P-value of < 0.05 was considered statistically significant.

### 2.5 Ethical considerations

This study adhered to the principles of the Declaration of Helsinki and followed the guidelines of the Strengthening the Reporting of Observational Studies in Epidemiology (STROBE). All data utilized in this study were de-identified to ensure the privacy and confidentiality of human subjects. The study protocol was reviewed and approved by the Ethics Committee of Peking Union Medical College Hospital, Chinese Academy of Medical Sciences (Approval No. SZ-9132). Written informed consent was obtained from all participants and/or their legal guardians prior to the study.

## 3 Results

### 3.1 Univariate and multivariate COX regression analysis

In this study, we employed Cox regression analysis to evaluate 12 potential factors for PFS and OS in HGG patients, including gender, TMZ-BEV interval, Karnofsky Performance Score (KPS), and 9 other variables. Univariate analysis revealed that pathological grade, extent of resection, MGMT status, and IDH status were associated with PFS. Multivariate analysis confirmed these factors as significant: pathological grade (HR 2.11, 95% CI 1.28-3.46, p=0.003), extent of resection (HR 2.19, 95% CI 1.38-3.49, p<0.001), MGMT status (HR 1.77, 95% CI 1.15-2.73, p=0.010), and IDH status (HR 1.67, 95% CI 1.06-2.61, p=0.026). Regarding OS, both univariate and multivariate analyses yielded consistent results, indicating that pathological grade (HR 1.91, 95% CI 1.13-3.23, p=0.015), extent of resection (HR 2.00, 95% CI 1.23-3.26, p=0.005), MGMT status (HR 1.94, 95% CI 1.23-3.06, p=0.005), and IDH status (HR 1.89, 95% CI 1.19-3.02, p=0.007) were significant predictors of OS. **Tables 2**, **3** present the Cox regression results for PFS and OS, respectively.

**Table 2:** Univariate and multivariate Cox regression analysis of progression-free survival.

Variables	N	Univariate		Multivariate	
		HR (95%CI)	p Value	(95%CI)	p Value
Age	103	1.00 (0.98; 1.02)	0.725		
Gender	103	1.02 (0.67; 1.56)	0.921		
TMZ cycle	92	0.86 (0.60; 1.22)	0.390		
TMZ interval	92	1.08 (0.89; 1.30)	0.445		
Steroid dose	103	1.01 (0.99; 1.03)	0.558		
KPS>70	58	0.85 (0.56; 1.30)	0.454		
Tumor>30ccm	49	1.48 (0.97; 2.25)	0.070		
Radiation therapy	103	1.23 (0.80; 1.89)	0.349		
Pathological grade	103	2.56 (1.58; 4.15)	<0.001	2.11 (1.28; 3.46)	0.003*
Gross resection	66	2.43 (1.55; 3.82)	<0.001	2.19 (1.38; 3.49)	<0.001*
MGMT status	103	1.69 (1.11; 2.58)	0.015	1.77 (1.15; 2.73)	0.010*
IDH status	103	2.09 (1.35; 3.25)	0.001	1.67 (1.06; 2.61)	0.026*

These variables with a P-value < 0.05 in the univariate analysis were included in the multivariate Cox regression analysis. *means P < 0.05.

HR, hazard ratio; CI, confidence interval; TMZ, temozolomide; KPS, karnofsky performance score; MGMT, O6-methylguanine-DNA methyltransferase; IDH, isocitrate dehydrogenase.

**Table 3:** Univariate and multivariate Cox regression analysis of overall survival.

Variables	N	Univariate		Multivariate	
		HR (95%CI)	p Value	(95%CI)	p Value
Age	103	1.00 (0.97; 1.02)	0.681		
Gender	103	0.97 (0.62; 1.52)	0.905		
TMZ cycle	92	0.85 (0.58; 1.23)	0.382		
TMZ interval	92	1.08 (0.88; 1.32)	0.477		
Steroid dose	103	1.01 (0.99; 1.04)	0.177		
KPS≥70	58	0.96 (0.61; 1.51)	0.865		
Tumor≥30ccm	49	1.44 (0.92; 2.25)	0.108		
Radiation therapy	103	1.25 (0.80; 1.98)	0.329		
Pathological grade	103	2.41 (1.44; 4.04)	<0.001	1.91 (1.13; 3.23)	0.015*
Gross resection	66	2.28 (1.42; 3.68)	<0.001	2.00 (1.23; 3.26)	0.005*
MGMT status	103	1.80 (1.15; 2.82)	0.010	1.94 (1.23; 3.06)	0.005*
IDH status	103	2.31 (1.46; 3.66)	<0.001	1.89 (1.19; 3.02)	0.007*

These variables with a P-value<0.05 in the univariate analysis were included in the multivariate Cox regression analysis. *means P < 0.05.

HR, hazard ratio; CI, confidence interval; TMZ, temozolomide; KPS, karnofsky performance score; MGMT, O6-methylguanine-DNA methyltransferase; IDH, isocitrate dehydrogenase.

### 3.2 Grouping based on pathological grade

**Figure 1:**
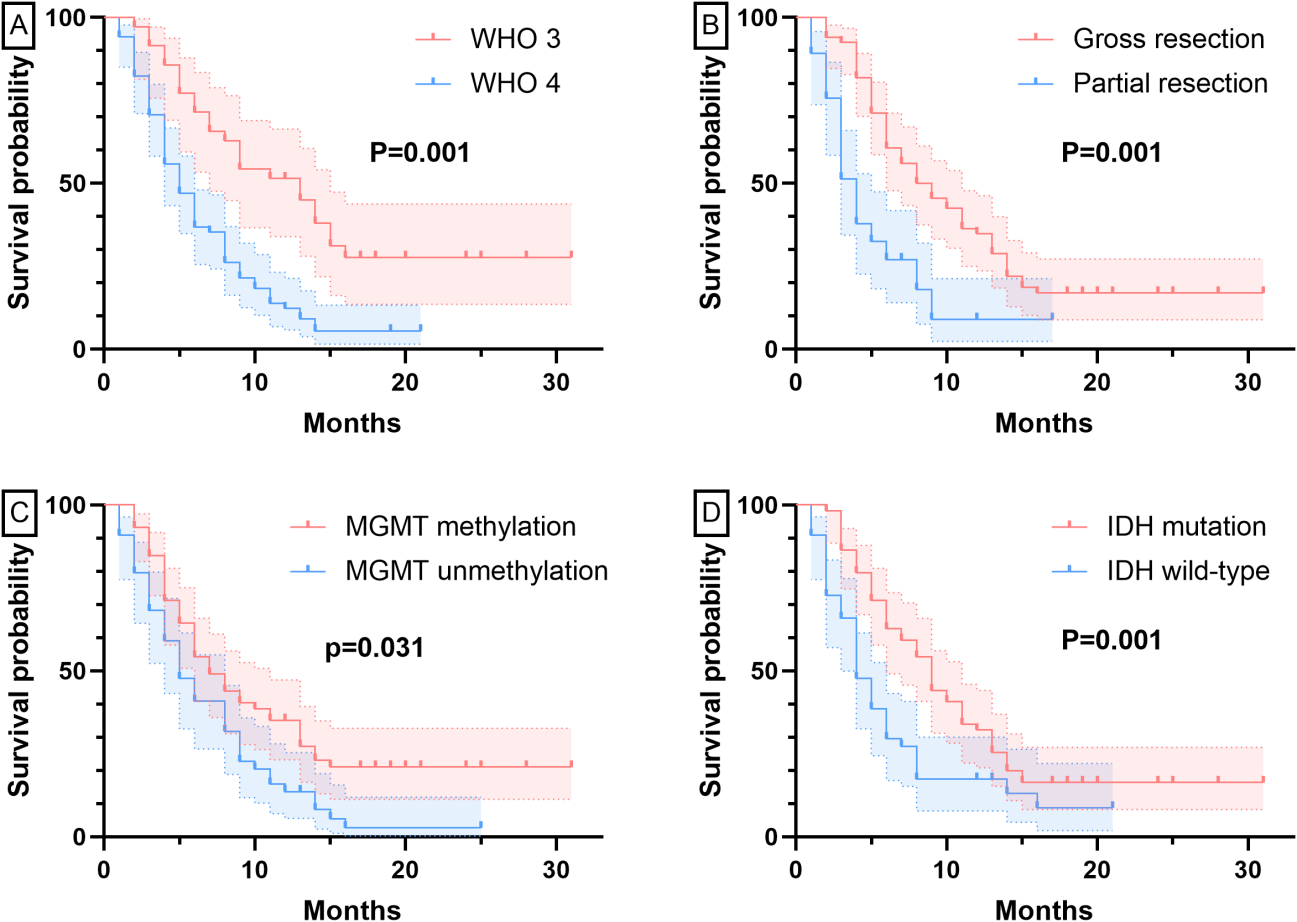
PFS curves grouped by different factors. Panel **(A)** Grouped by pathological grade; Panel **(B)** Grouped by extent of resection; Panel **(C)** Grouped by MGMT status; Panel **(D)** Grouped by IDH status.

**Figure 2:**
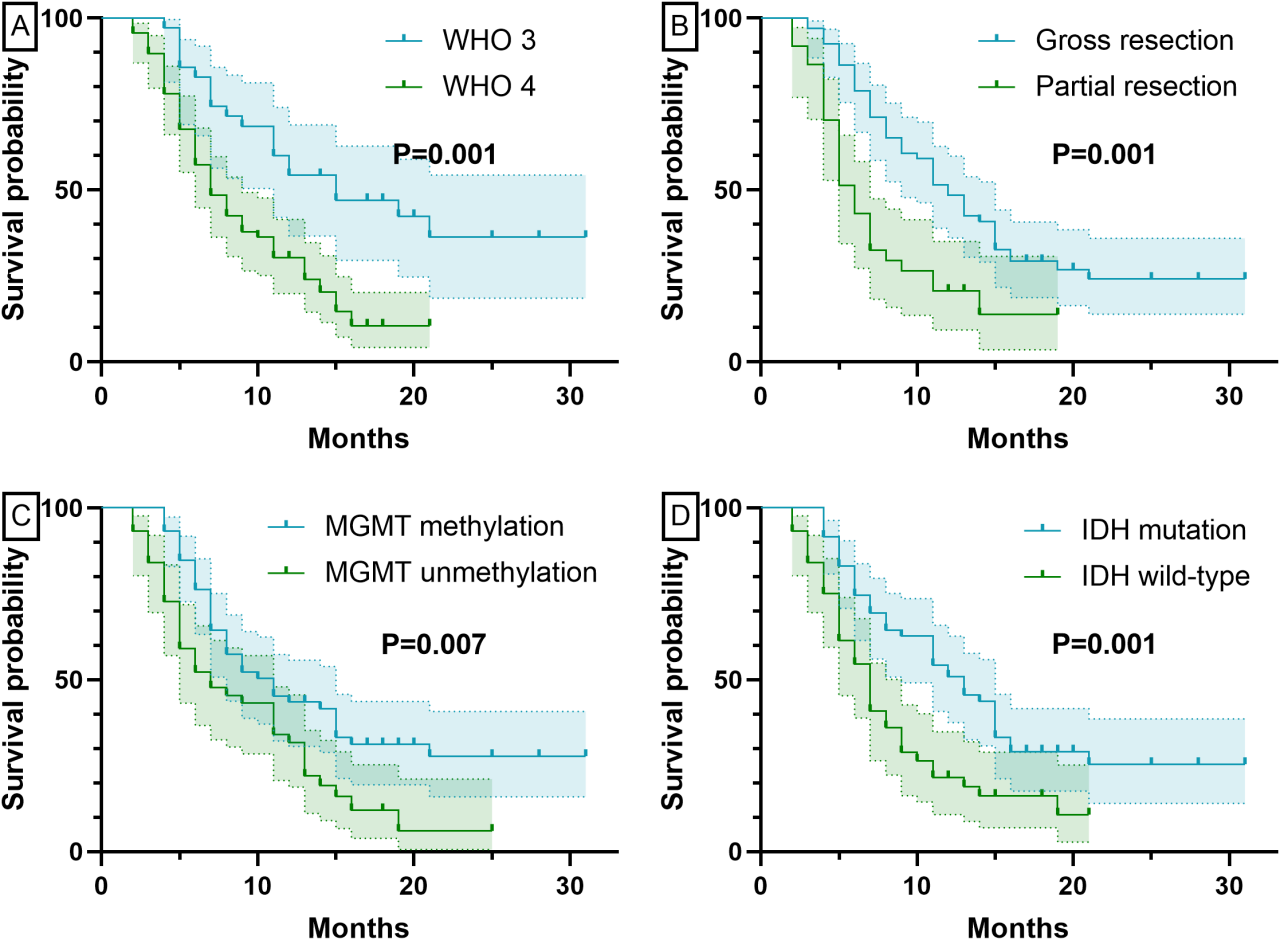
OS curves grouped by different factors. Panel **(A)** Grouped by pathological grade; Panel **(B)** Grouped by extent of resection; Panel **(C)** Grouped by MGMT status; Panel **(D)** Grouped by IDH status.

At six-month follow-up, BEV-treated patients with WHO grade 3 tumors (n=35) demonstrated significantly better outcomes than grade 4 cases (n=68). Tumor progression occurred in 11 grade 3 versus 41 grade 4 patients (31.4% vs 60.3%, P=0.006), with higher OR (82.9% [22 CR, 7 PR] vs 47.1% [24 CR, 8 PR], P<0.001) and fewer PD cases (5.7% vs 20.6%). Grade 3 tumors also showed superior survival (PFS: 12.23 ± 7.61 vs 6.44 ± 4.61 months; OS: 14.29 ± 7.43 vs 8.78 ± 5.02 months, both P<0.001). **Figures 1**, **2** show the PFS and OS curves of HGG patients grouped by various factors, respectively.

### 3.3 Grouping based on extent of resection

At six-month follow-up, patients with gross resection (n=66) showed significantly better outcomes than partial resection cases (n=37). Tumor progression occurred in 24 gross versus 28 partial resection patients (36.4% vs 75.7%, P<0.001), with higher OR (75.8% [39 CR, 11 PR] vs 29.7% [7 CR, 4 PR], P<0.001) and fewer PD cases (7.6% vs 29.7%). The gross resection group also demonstrated superior survival (PFS: 10.36 ± 6.78 vs 4.92 ± 3.62 months; OS: 12.68 ± 6.70 vs 7.03 ± 4.06 months, both P<0.001).

### 3.4 Grouping based on MGMT status

Patients were stratified into MGMT methylated (n=59) and unmethylated (n=44) groups. At six-month follow-up, tumor progression occurred in 24 methylated versus 28 unmethylated patients, with significantly higher PFS rates (59.3% vs 36.4%, P=0.021) and OR (69.5% [33 CR, 8 PR] vs 45.5% [13 CR, 7 PR], P=0.014). The methylated group showed better survival outcomes (PFS: 9.64 ± 7.02 vs 6.75 ± 5.05 months; OS: 12.05 ± 6.86 vs 8.77 ± 5.43 months), with comparable PD rates (8 vs 8 cases) but fewer SD cases (10 vs 16).

### 3.5 Grouping based on IDH status

At 6 months post-BEV treatment, tumor recurrence occurred in 52 of 103 patients (22/61 IDH-mutated vs. 30/42 IDH-wildtype). The IDH-mutated group exhibited significantly higher PFS rates (63.9% vs. 28.6%, *P* < 0.001) and OR (73.8% [36 CR, 9 PR] vs. 38.1% [10 CR, 6 PR], *P* < 0.001), with fewer cases of SD/PD (7/9 vs. 19/7). Survival analysis further revealed superior outcomes for IDH-mutated patients (PFS: 10.43 ± 6.69 vs. 5.48 ± 4.60 months; OS: 12.80 ± 6.72 vs. 7.52 ± 4.58 months, both *P* < 0.001).

### 3.6 Grouping based on MGMT and IDH status

Patients were stratified by MGMT methylation status and further categorized by IDH mutation status into four subgroups: MGMT methylated-IDH mutant, MGMT methylated-IDH wildtype, MGMT unmethylated-IDH mutant, and MGMT unmethylated-IDH wildtype. At six-month follow-up, tumor progression distribution revealed 11 IDH mutant and 13 IDH wildtype cases in the MGMT methylated group (total 24 progressions), compared to 11 IDH mutant and 17 IDH wildtype cases in the MGMT unmethylated group (total 28 progressions).

**Figure 3:**
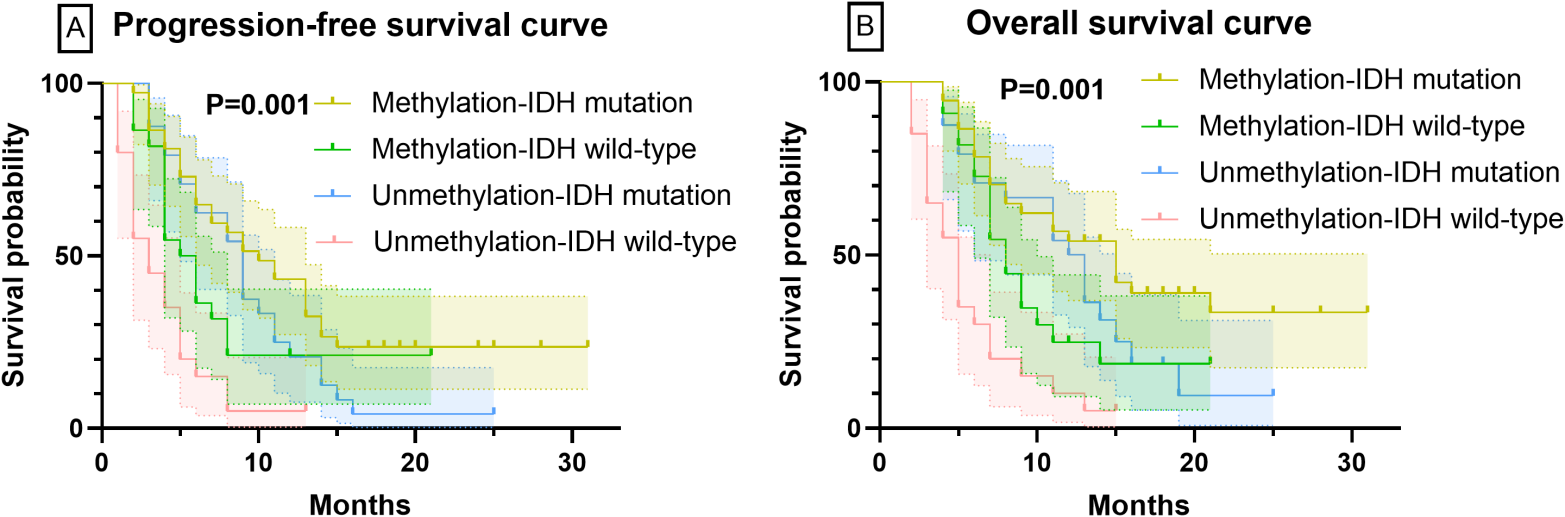
PFS and OS curves based on the combined status of MGMT and IDH. Panel **(A)** PFS curves for patients with different biomarker statuses. Panel **(B)** OS curves for patients with different biomarker statuses.

Treatment response analysis demonstrated significantly different OR rates across subgroups: MGMT methylated-IDH mutant (81.1%, 30/37 patients) showed the highest response, followed by MGMT unmethylated-IDH mutant (62.5%, 15/24), MGMT methylated-IDH wildtype (50.0%, 11/22), and MGMT unmethylated-IDH wildtype (25.0%, 5/20) (all P<0.001). Survival outcomes exhibited a progressive deterioration pattern: PFS durations were 11.27 ± 7.46 months (methylated-mutant) > 9.13 ± 5.19 (unmethylated-mutant) > 6.91 ± 5.33 (methylated-wildtype) > 3.90 ± 3.06 months (unmethylated-wildtype), while OS followed a similar trend (13.76 ± 7.39 > 11.33 ± 5.36 > 9.18 ± 4.75 > 5.70 ± 3.70 months, all P<0.001). The corresponding survival curves are presented in **Figure 3**.

### 3.7 Grouping based on radiotherapy

At six-month follow-up, tumor progression occurred in 35 EBRT-treated versus 17 IMRT-treated patients (P=0.627). Treatment responses showed comparable OR between groups (EBRT: 59.7% [29 CR, 11 PR] vs IMRT: 58.3% [17 CR, 4 PR], P=0.893), though EBRT had more SD cases (18 vs 8). Survival outcomes were similar between modalities (PFS: 8.76 ± 6.96 vs 7.75 ± 5.21 months; OS: 10.85 ± 6.73 vs 10.28 ± 6.03 months), with no statistically significant differences observed.

### 3.8 Adverse drug reactions

The adverse reactions associated with BEV encompass a range of symptoms including hemorrhage, headache, hypertension, hematologic toxicity, thrombosis, proteinuria, gastrointestinal perforation, delayed wound healing, sepsis, and nephrotic syndrome (18). In the present study, headache was the most frequently reported adverse reaction, occurring in a total of 16 patients. Additionally, 11 patients developed new-onset hypertension following BEV administration, 6 patients experienced gingival hemorrhage, and 5 patients exhibited mild hematologic toxicity. Notably, no severe adverse reactions necessitating discontinuation of BEV therapy were observed.

## 4 Discussion

HGG remains a devastating diagnosis, with standard treatment comprising maximal safe resection followed by 6 weeks of concurrent radiotherapy and TMZ, then 6 months of adjuvant TMZ (19, 20). BEV, an anti-angiogenic agent, has been shown to improve PFS and health-related quality of life in HGG patients, supporting its use as adjuvant therapy (21–23). However, whether BEV’s efficacy is modulated by chemotherapeutic agents like TMZ remains unknown. Although MGMT promoter methylation and IDH mutation status are established prognostic factors in HGG, their predictive role in BEV treatment response remains uncharacterized. This study focused on a chemotherapy-limited cohort (patients receiving no chemotherapy or substandard TMZ doses) to evaluate BEV efficacy in this setting and its association with MGMT/IDH status.

Our study data reveal that WHO grade 4 patients exhibited significantly shorter PFS and OS compared to grade 3 patients, which aligns with clinical observations in the latest WHO classification of central nervous system tumors (24). The prognostic significance of pathological grading manifests in several aspects: primarily, higher-grade tumors display more prominent microvascular proliferation and necrotic features, histological characteristics directly associated with aggressive biological behavior; secondly, increasing pathological grade correlates with markedly elevated tumor cell proliferation activity and genomic instability (25–27). Notably, even among IDH-mutant and MGMT methylated HGG, WHO grade 4 patients still demonstrated significantly worse prognosis than grade 3 patients, indicating that pathological grading maintains prognostic value independent of these molecular features (28). Our multivariate analysis further confirmed that after adjusting for both MGMT methylation status and IDH mutation status, pathological grading remained an independent predictor for both PFS and OS.

Clinically, maximal safe resection improves prognosis through three primary mechanisms: (1) direct tumor burden reduction that delays recurrence; (2) diminished pro-angiogenic factor secretion from residual tumor cells; and (3) creation of a more favorable microenvironment for subsequent chemo-radiotherapy (29). Notably, in the anti-angiogenic therapy era, gross resection gains additional significance—our subgroup analysis showed that BEV-treated patients with gross resection achieved more durable responses than those with partial resection (30). This effect likely reflects the vascular microenvironment remodeling induced by complete surgical removal (31). Thus, even in the molecular marker-guided therapy era, the extent of surgical resection remains a crucial prognostic factor worthy of emphasis.

A study involving 191 patients reported no association between MGMT status and prognosis, although MGMT status was unknown in 63% of the cohort (32). Another investigation of 92 recurrent HGG patients observed higher MGMT methylation rates in long-term responders; however, concomitant administration of TMZ or fotemustine may have confounded these results (33). In contrast to these studies, our research evaluated the prognostic value of MGMT in a rigorously controlled cohort with low chemotherapy intensity, thereby minimizing therapeutic interference. Specifically, the MGMT-methylated group demonstrated significantly superior PFS and OS along with higher objective response (OR) rates, suggesting that MGMT methylation may potentiate BEV efficacy - a finding consistent with previous reports (34, 35).

Nevertheless, CR rates require cautious interpretation for two key reasons: first, BEV may reduce MRI contrast enhancement through decreased vascular permeability rather than true tumor regression; second, the frequent pseudoprogression in MGMT-methylated tumors that shows rapid improvement with BEV could be misinterpreted as therapeutic response (36). Thus, the observed CR rates may partially reflect BEV’s vascular modulation effects rather than genuine tumor regression. Importantly, the persistent survival advantage in MGMT-methylated patients suggests that this biomarker may enhance BEV efficacy through mechanisms involving regulation of VEGF-dependent angiogenesis.

Our study demonstrated that IDH-mutant patients exhibited superior PFS, OS, and OR rates compared to their IDH-wildtype counterparts, with this difference remaining statistically significant after adjustment for MGMT status. IDH-mutant tumors typically display reduced angiogenic activity and a more stabilized microenvironment, which may prolong the vascular normalization effects of BEV. Furthermore, IDH mutation is associated with metabolic reprogramming (e.g., 2-HG accumulation) that potentially suppresses pro-angiogenic factors (such as HIF-1α expression), thereby potentiating the anti-angiogenic activity of BEV (37, 38). Notably, IDH mutation maintained its prognostic value even among MGMT-unmethylated patients.

Finally, to exclude the potential interference of MGMT methylation on IDH, we further categorized patients into four subgroups: MGMT-methylated with IDH mutation, MGMT-methylated with IDH wild-type, MGMT-unmethylated with IDH mutation, and MGMT-unmethylated with IDH wild-type. The results showed that regardless of MGMT methylation status, patients with IDH mutations had superior PFS, OS, and treatment response compared to those with the IDH wild-type. In conclusion, we believe that HGG patients with both MGMT methylation and IDH mutations may derive greater benefit from BEV treatment.

The comparable outcomes between EBRT and IMRT subgroups suggest that BEV efficacy in high-grade glioma may be independent of radiotherapy technique when using standard dose regimens. This observation aligns with the proposed vascular normalization mechanism of anti-angiogenic therapy, where the biological effects of radiation dose rather than delivery technique appear predominant for therapeutic synergy (39). Clinical data from the AVAglio trial similarly showed consistent BEV benefit across different radiotherapy approaches in newly diagnosed glioblastoma (40). While these findings support the robustness of BEV effects across conventional radiotherapy modalities, further investigation is warranted to evaluate potential interactions with specific tumor molecular subtypes or unusual fractionation schemes.

By excluding the potential interference of chemotherapy agents, our study provides that HGG patients harboring both MGMT methylation and IDH mutations are likely to derive greater benefit from BEV treatment. Despite these significant findings, it is important to acknowledge some potential limitations of our study. Firstly, as a single-center study, the results may not be fully representative. Secondly, due to the limited number of patients who discontinued TMZ in clinical practice, our study had a relatively small sample size. Finally, our study was unable to overcome the inherent limitations of a retrospective study. Future multicenter, large-scale, prospective studies are necessary to address these limitations and further validate our findings.

## 5 Conclusion

After excluding the interference of chemotherapy drugs, our study demonstrates that HGG patients with both MGMT methylation and IDH mutations are likely to derive greater benefit from BEV treatment.

## Data Availability

The original contributions presented in the study are publicly available. This data can be found here: 10.5281/zenodo.16900830, https://zenodo.org/records/16900831.
